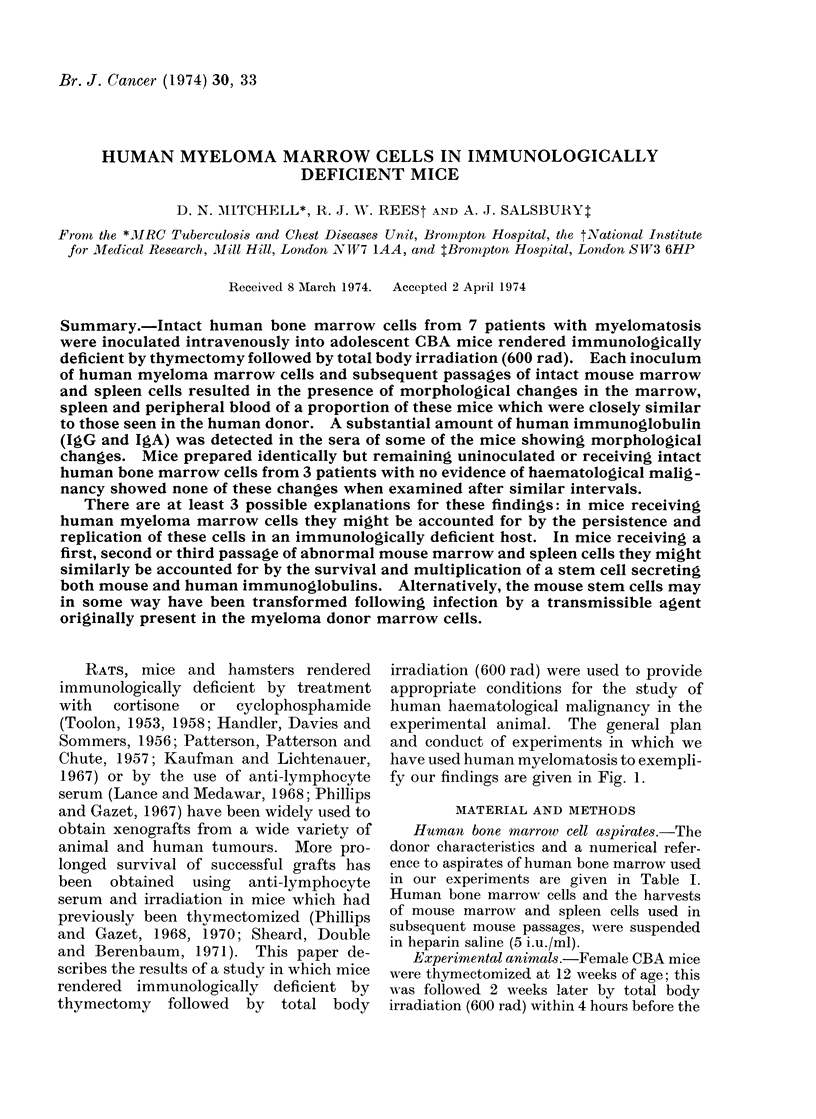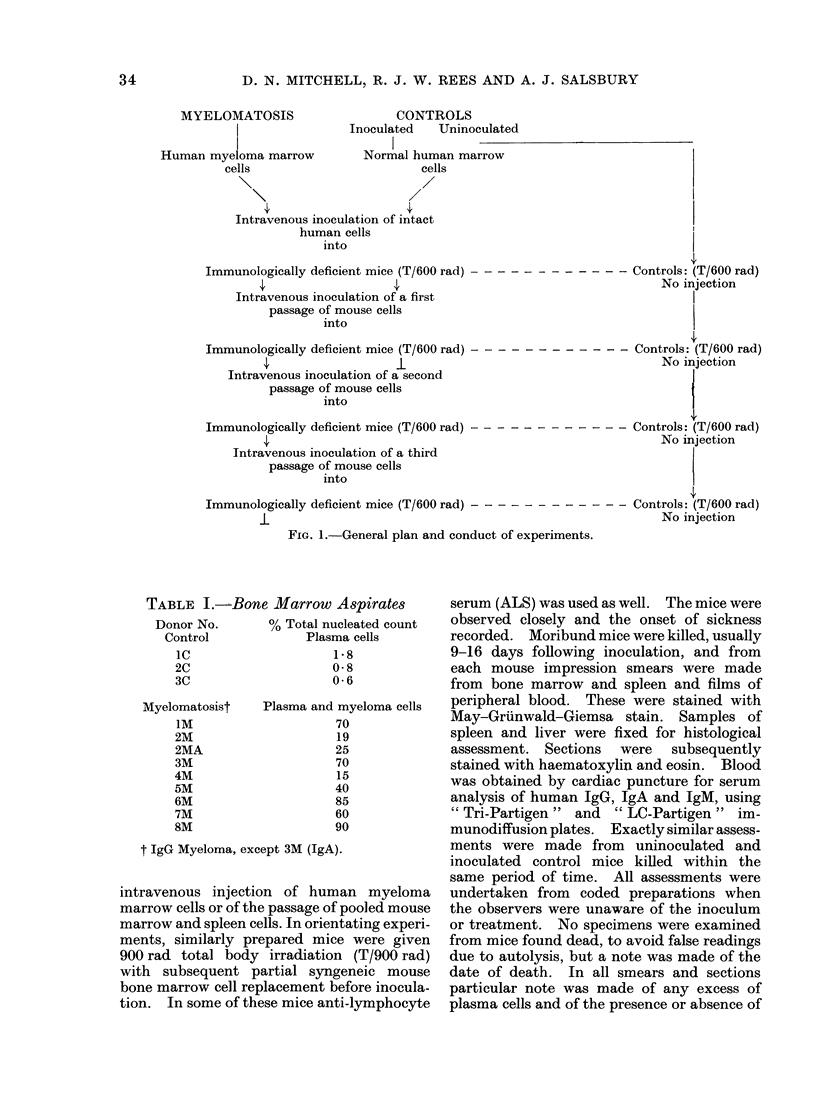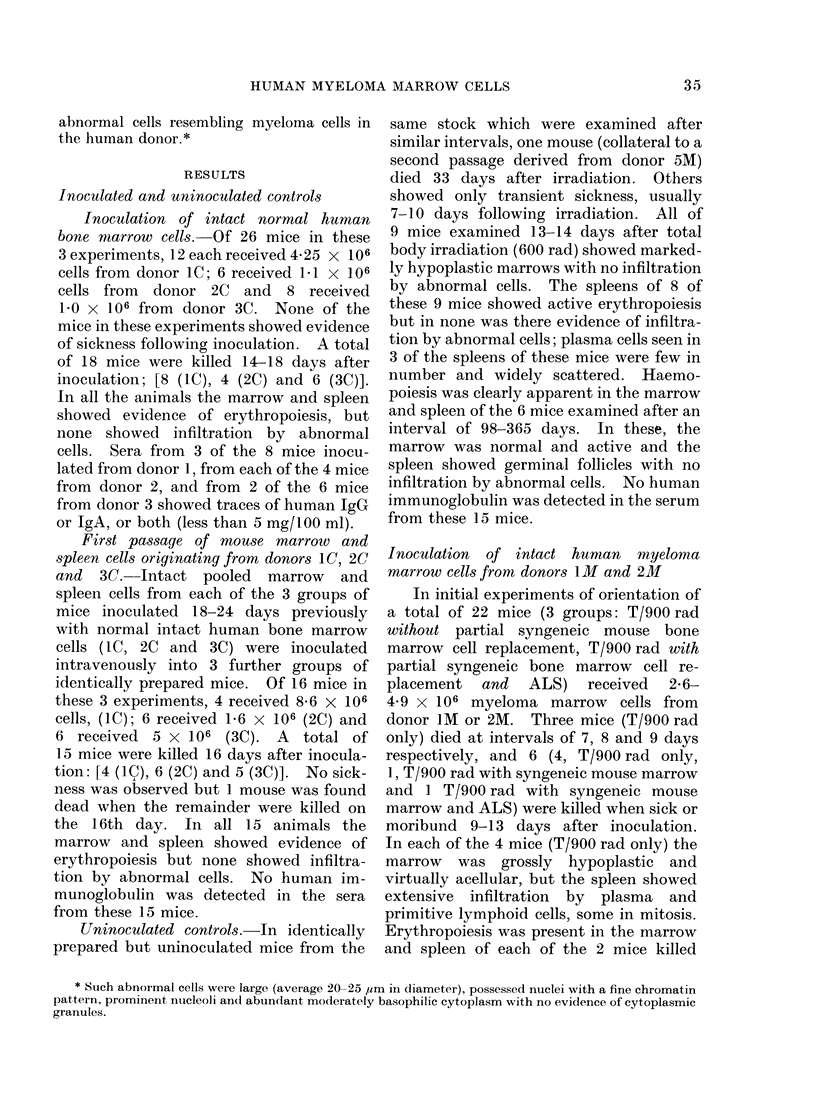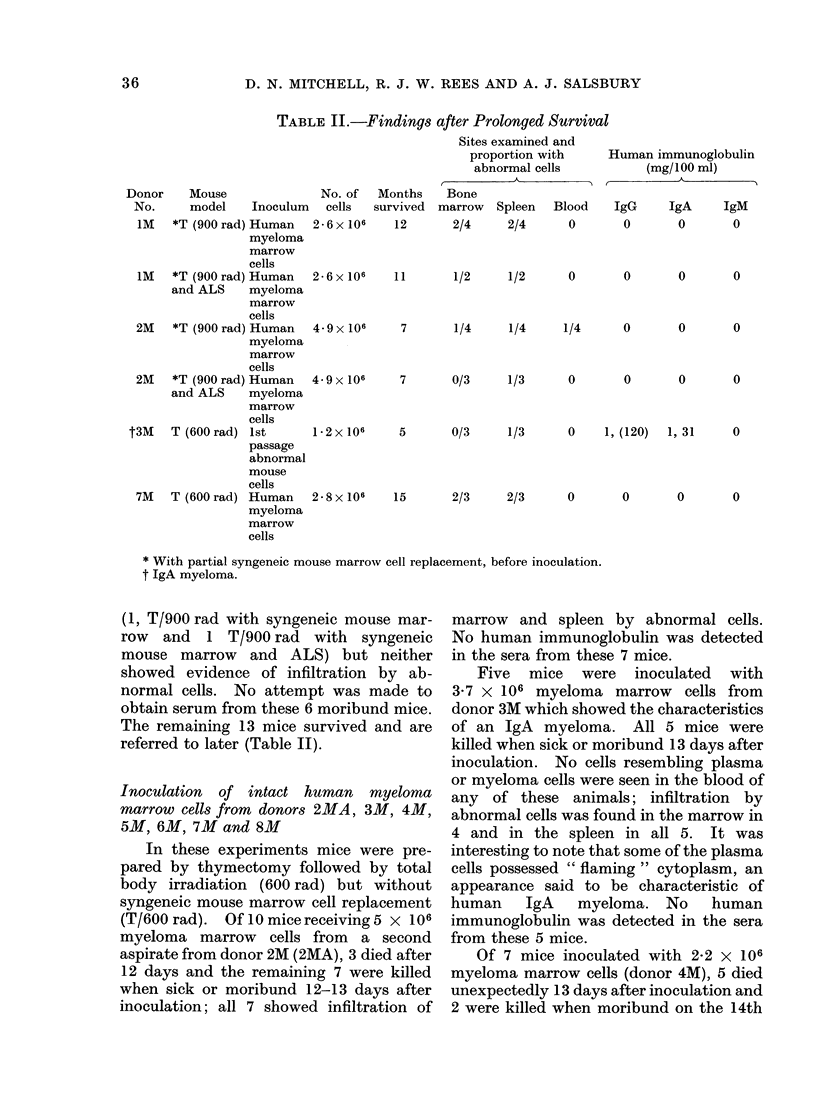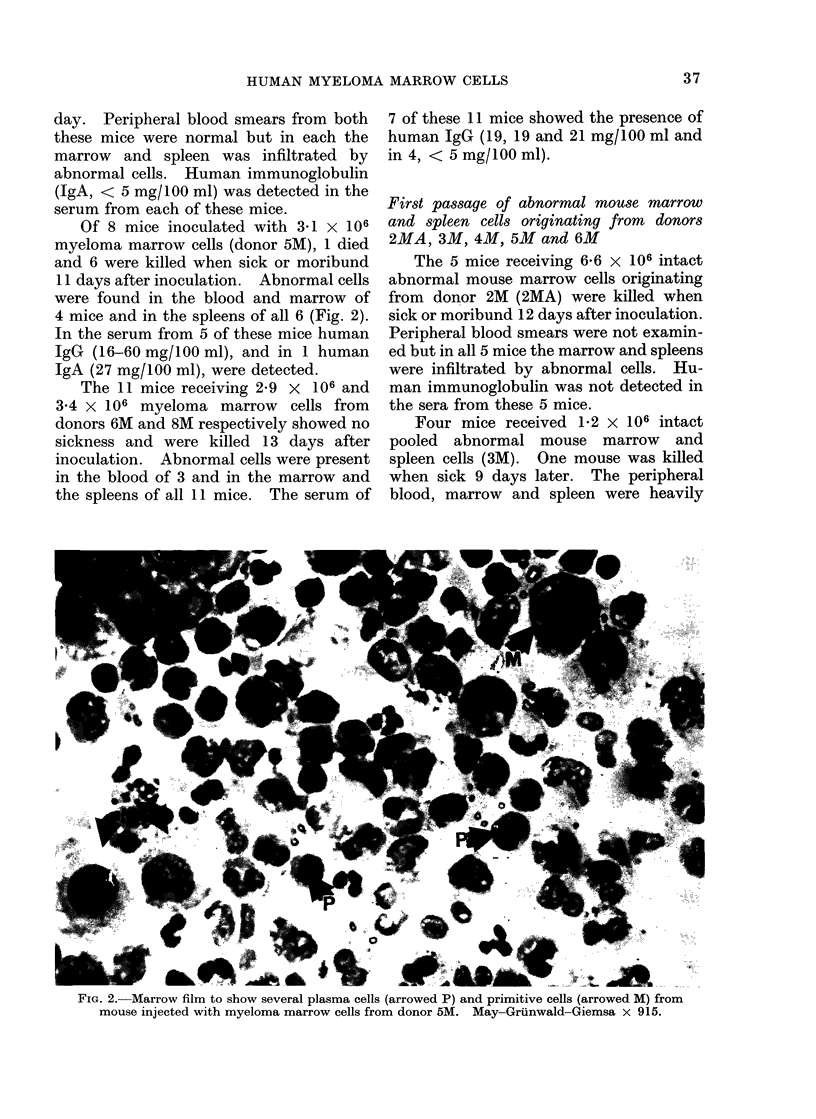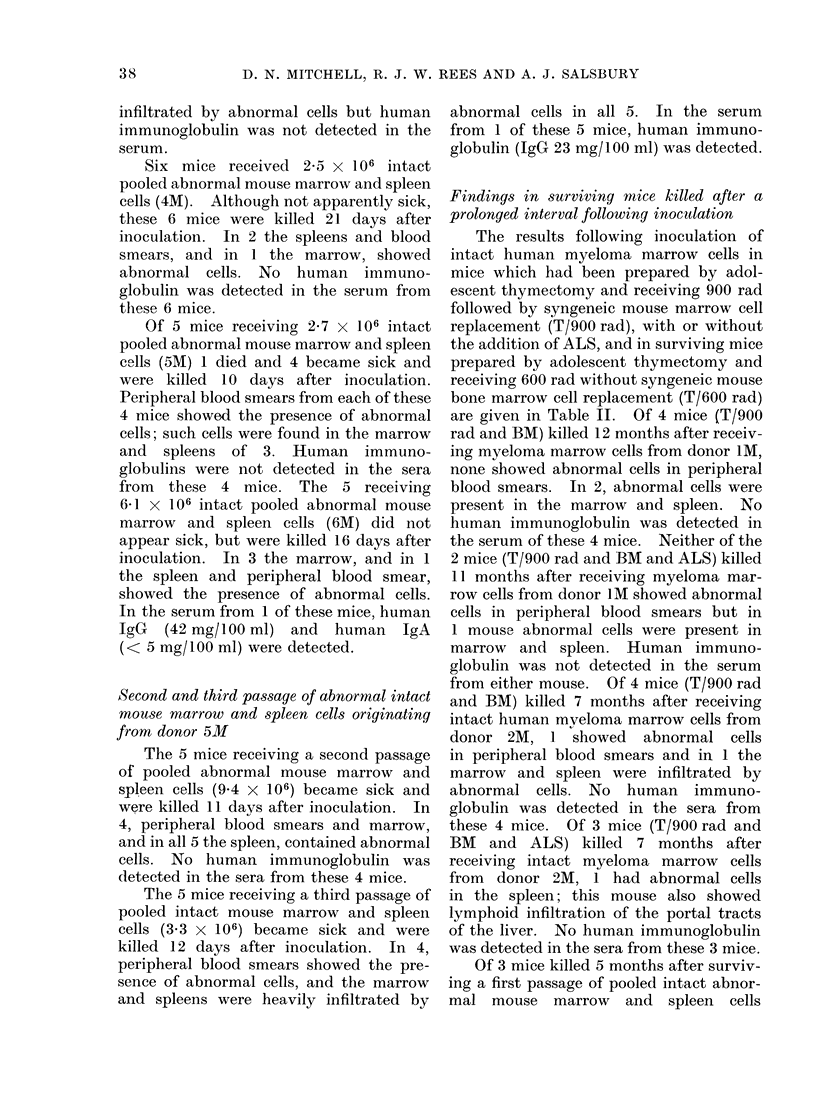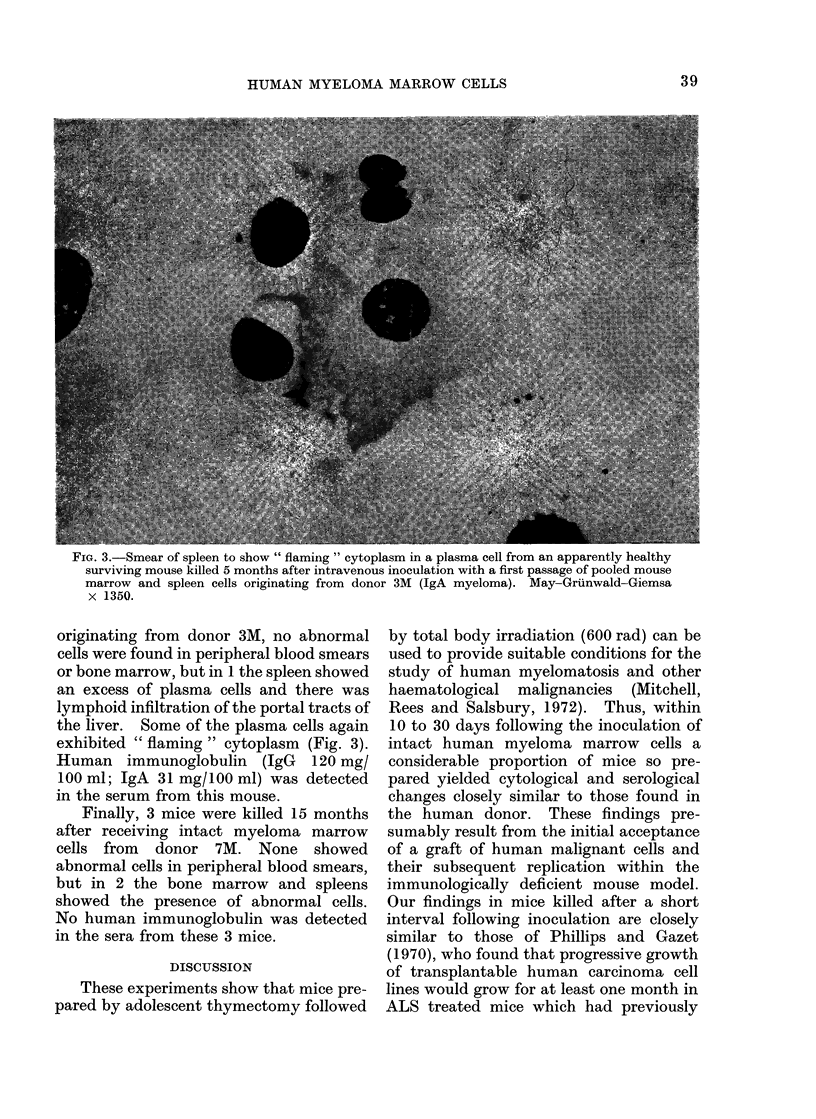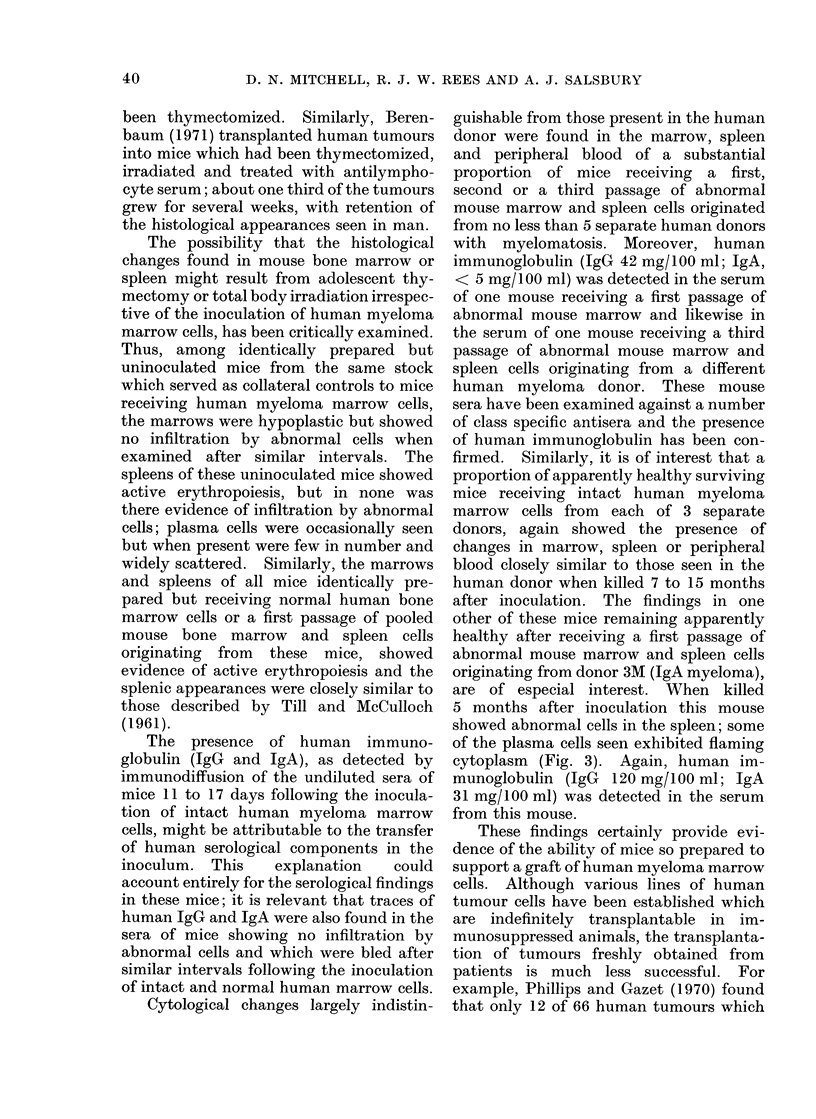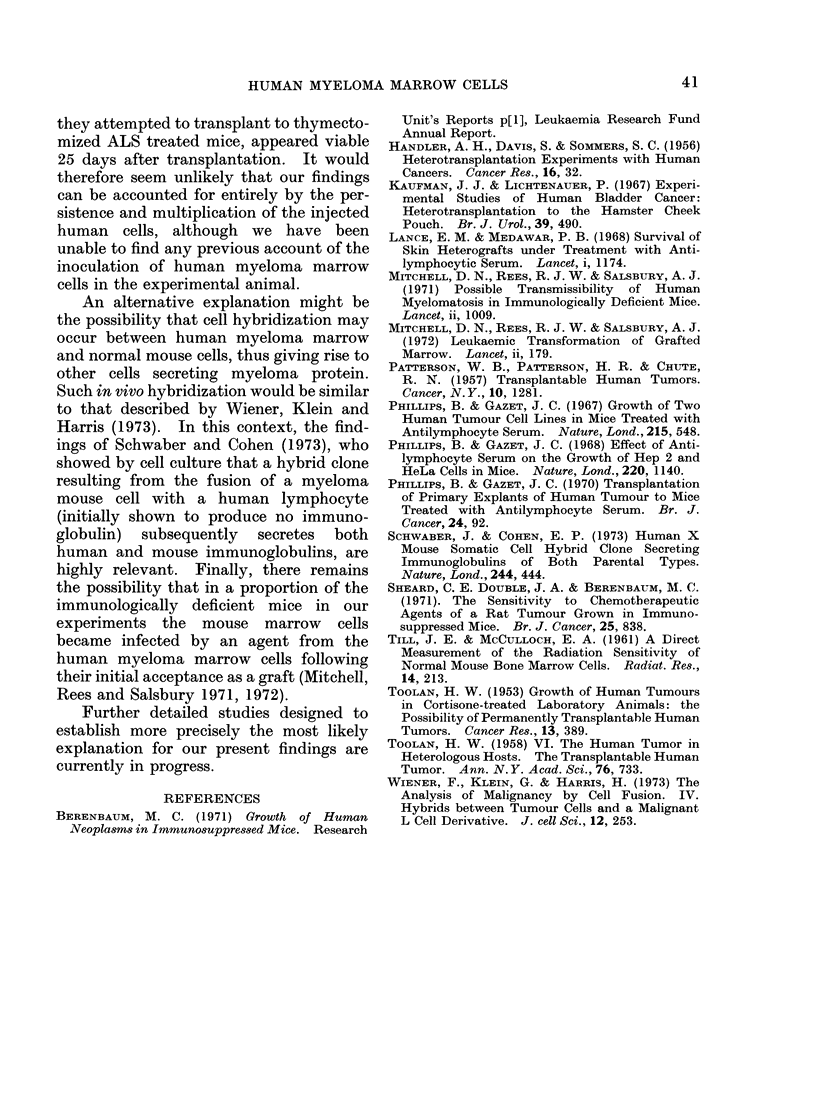# Human Myeloma Marrow Cells in Immunologically Deficient Mice

**DOI:** 10.1038/bjc.1974.110

**Published:** 1974-07

**Authors:** D. N. Mitchell, R. J. W. Rees, A. J. Salsbury

## Abstract

**Images:**


*Br. J. Cancer* (1974) **30**, 33

### HUMAN MYELOMA MARROW CELLS IN IMMUNOLOGICALLY DEFICIENT MICE

D. N. MITCHELL*, R. J. W. REES† AND A. J. SALSBURY‡

*From the *MRC Tuberculosis and Chest Diseases Unit, Brompton Hospital, the †National Institute for Medical Research, Mill Hill, London NW7 1AA, and ‡Brompton Hospital, London SW3 6HP*

Received 8 March 1974. Accepted 2 April 1974

**Summary.—Intact human bone marrow cells from 7 patients with myelomatosis were inoculated intravenously into adolescent CBA mice rendered immunologically deficient by thymectomy followed by total body irradiation (600 rad). Each inoculum of human myeloma marrow cells and subsequent passages of intact mouse marrow and spleen cells resulted in the presence of morphological changes in the marrow, spleen and peripheral blood of a proportion of these mice which were closely similar to those seen in the human donor. A substantial amount of human immunoglobulin (IgG and IgA) was detected in the sera of some of the mice showing morphological changes. Mice prepared identically but remaining uninoculated or receiving intact human bone marrow cells from 3 patients with no evidence of haematological malignancy showed none of these changes when examined after similar intervals.**

**There are at least 3 possible explanations for these findings: in mice receiving human myeloma marrow cells they might be accounted for by the persistence and replication of these cells in an immunologically deficient host. In mice receiving a first, second or third passage of abnormal mouse marrow and spleen cells they might similarly be accounted for by the survival and multiplication of a stem cell secreting both mouse and human immunoglobulins. Alternatively, the mouse stem cells may in some way have been transformed following infection by a transmissible agent originally present in the myeloma donor marrow cells.**

RATS, mice and hamsters rendered immunologically deficient by treatment with cortisone or cyclophosphamide (Toolon, 1953, 1958; Handler, Davies and Sommers, 1956; Patterson, Patterson and Chute, 1957; Kaufman and Lichtenauer, 1967) or by the use of anti-lymphocyte serum (Lance and Medawar, 1968; Phillips and Gazet, 1967) have been widely used to obtain xenografts from a wide variety of animal and human tumours. More prolonged survival of successful grafts has been obtained using anti-lymphocyte serum and irradiation in mice which had previously been thymectomized (Phillips and Gazet, 1968, 1970; Sheard, Double and Berenbaum, 1971). This paper describes the results of a study in which mice rendered immunologically deficient by thymectomy followed by total body irradiation (600 rad) were used to provide appropriate conditions for the study of human haematological malignancy in the experimental animal. The general plan and conduct of experiments in which we have used human myelomatosis to exemplify our findings are given in Fig. 1.

### MATERIAL AND METHODS

*Human bone marrow cell aspirates.*—The donor characteristics and a numerical reference to aspirates of human bone marrow used in our experiments are given in Table I. Human bone marrow cells and the harvests of mouse marrow and spleen cells used in subsequent mouse passages, were suspended in heparin saline (5 i.u./ml).

*Experimental animals.*—Female CBA mice were thymectomized at 12 weeks of age; this was followed 2 weeks later by total body irradiation (600 rad) within 4 hours before the

FIG. 1.—General plan and conduct of experiments.

TABLE I.—*Bone Marrow Aspirates*

| Donor No. | % Total nucleated count |
|---|---|
| Control | Plasma cells |
| 1C | 1·8 |
| 2C | 0·8 |
| 3C | 0·6 |
| Myelomatosis† | Plasma and myeloma cells |
| 1M | 70 |
| 2M | 19 |
| 2MA | 25 |
| 3M | 70 |
| 4M | 15 |
| 5M | 40 |
| 6M | 85 |
| 7M | 60 |
| 8M | 90 |

† IgG Myeloma, except 3M (IgA).

intravenous injection of human myeloma marrow cells or of the passage of pooled mouse marrow and spleen cells. In orientating experiments, similarly prepared mice were given 900 rad total body irradiation (T/900 rad) with subsequent partial syngeneic mouse bone marrow cell replacement before inoculation. In some of these mice anti-lymphocyte serum (ALS) was used as well. The mice were observed closely and the onset of sickness recorded. Moribund mice were killed, usually 9–16 days following inoculation, and from each mouse impression smears were made from bone marrow and spleen and films of peripheral blood. These were stained with May–Grünwald–Giemsa stain. Samples of spleen and liver were fixed for histological assessment. Sections were subsequently stained with haematoxylin and eosin. Blood was obtained by cardiac puncture for serum analysis of human IgG, IgA and IgM, using " Tri-Partigen " and " LC-Partigen " immunodiffusion plates. Exactly similar assessments were made from uninoculated and inoculated control mice killed within the same period of time. All assessments were undertaken from coded preparations when the observers were unaware of the inoculum or treatment. No specimens were examined from mice found dead, to avoid false readings due to autolysis, but a note was made of the date of death. In all smears and sections particular note was made of any excess of plasma cells and of the presence or absence of abnormal cells resembling myeloma cells in the human donor.*

RESULTS

*Inoculated and uninoculated controls*

*Inoculation of intact normal human bone marrow cells.*—Of 26 mice in these 3 experiments, 12 each received $4{\cdot}25 \times 10^6$ cells from donor 1C; 6 received $1{\cdot}1 \times 10^6$ cells from donor 2C and 8 received $1{\cdot}0 \times 10^6$ from donor 3C. None of the mice in these experiments showed evidence of sickness following inoculation. A total of 18 mice were killed 14–18 days after inoculation; [8 (1C), 4 (2C) and 6 (3C)]. In all the animals the marrow and spleen showed evidence of erythropoiesis, but none showed infiltration by abnormal cells. Sera from 3 of the 8 mice inoculated from donor 1, from each of the 4 mice from donor 2, and from 2 of the 6 mice from donor 3 showed traces of human IgG or IgA, or both (less than 5 mg/100 ml).

*First passage of mouse marrow and spleen cells originating from donors 1C, 2C and 3C.*—Intact pooled marrow and spleen cells from each of the 3 groups of mice inoculated 18–24 days previously with normal intact human bone marrow cells (1C, 2C and 3C) were inoculated intravenously into 3 further groups of identically prepared mice. Of 16 mice in these 3 experiments, 4 received $8{\cdot}6 \times 10^6$ cells, (1C); 6 received $1{\cdot}6 \times 10^6$ (2C) and 6 received $5 \times 10^6$ (3C). A total of 15 mice were killed 16 days after inoculation: [4 (1C), 6 (2C) and 5 (3C)]. No sickness was observed but 1 mouse was found dead when the remainder were killed on the 16th day. In all 15 animals the marrow and spleen showed evidence of erythropoiesis but none showed infiltration by abnormal cells. No human immunoglobulin was detected in the sera from these 15 mice.

*Uninoculated controls.*—In identically prepared but uninoculated mice from the same stock which were examined after similar intervals, one mouse (collateral to a second passage derived from donor 5M) died 33 days after irradiation. Others showed only transient sickness, usually 7–10 days following irradiation. All of 9 mice examined 13–14 days after total body irradiation (600 rad) showed markedly hypoplastic marrows with no infiltration by abnormal cells. The spleens of 8 of these 9 mice showed active erythropoiesis but in none was there evidence of infiltration by abnormal cells; plasma cells seen in 3 of the spleens of these mice were few in number and widely scattered. Haemopoiesis was clearly apparent in the marrow and spleen of the 6 mice examined after an interval of 98–365 days. In these, the marrow was normal and active and the spleen showed germinal follicles with no infiltration by abnormal cells. No human immunoglobulin was detected in the serum from these 15 mice.

*Inoculation of intact human myeloma marrow cells from donors 1M and 2M*

In initial experiments of orientation of a total of 22 mice (3 groups: T/900 rad *without* partial syngeneic mouse bone marrow cell replacement, T/900 rad *with* partial syngeneic bone marrow cell replacement *and* ALS) received $2{\cdot}6$–$4{\cdot}9 \times 10^6$ myeloma marrow cells from donor 1M or 2M. Three mice (T/900 rad only) died at intervals of 7, 8 and 9 days respectively, and 6 (4, T/900 rad only, 1, T/900 rad with syngeneic mouse marrow and 1 T/900 rad with syngeneic mouse marrow and ALS) were killed when sick or moribund 9–13 days after inoculation. In each of the 4 mice (T/900 rad only) the marrow was grossly hypoplastic and virtually acellular, but the spleen showed extensive infiltration by plasma and primitive lymphoid cells, some in mitosis. Erythropoiesis was present in the marrow and spleen of each of the 2 mice killed

* Such abnormal cells were large (average 20–25 μm in diameter), possessed nuclei with a fine chromatin pattern, prominent nucleoli and abundant moderately basophilic cytoplasm with no evidence of cytoplasmic granules.

TABLE II.—*Findings after Prolonged Survival*

| Donor No. | Mouse model | Inoculum | No. of cells | Months survived | Sites examined and proportion with abnormal cells: Bone marrow | Spleen | Blood | Human immunoglobulin (mg/100 ml): IgG | IgA | IgM |
|---|---|---|---|---|---|---|---|---|---|---|
| 1M | *T (900 rad) | Human myeloma marrow cells | $2 \cdot 6 \times 10^6$ | 12 | 2/4 | 2/4 | 0 | 0 | 0 | 0 |
| 1M | *T (900 rad) and ALS | Human myeloma marrow cells | $2 \cdot 6 \times 10^6$ | 11 | 1/2 | 1/2 | 0 | 0 | 0 | 0 |
| 2M | *T (900 rad) | Human myeloma marrow cells | $4 \cdot 9 \times 10^6$ | 7 | 1/4 | 1/4 | 1/4 | 0 | 0 | 0 |
| 2M | *T (900 rad) and ALS | Human myeloma marrow cells | $4 \cdot 9 \times 10^6$ | 7 | 0/3 | 1/3 | 0 | 0 | 0 | 0 |
| †3M | T (600 rad) | 1st passage abnormal mouse cells | $1 \cdot 2 \times 10^6$ | 5 | 0/3 | 1/3 | 0 | 1, (120) | 1, 31 | 0 |
| 7M | T (600 rad) | Human myeloma marrow cells | $2 \cdot 8 \times 10^6$ | 15 | 2/3 | 2/3 | 0 | 0 | 0 | 0 |

* With partial syngeneic mouse marrow cell replacement, before inoculation.
† IgA myeloma.

(1, T/900 rad with syngeneic mouse marrow and 1 T/900 rad with syngeneic mouse marrow and ALS) but neither showed evidence of infiltration by abnormal cells. No attempt was made to obtain serum from these 6 moribund mice. The remaining 13 mice survived and are referred to later (Table II).

*Inoculation of intact human myeloma marrow cells from donors 2MA, 3M, 4M, 5M, 6M, 7M and 8M*

In these experiments mice were prepared by thymectomy followed by total body irradiation (600 rad) but without syngeneic mouse marrow cell replacement (T/600 rad). Of 10 mice receiving $5 \times 10^6$ myeloma marrow cells from a second aspirate from donor 2M (2MA), 3 died after 12 days and the remaining 7 were killed when sick or moribund 12–13 days after inoculation; all 7 showed infiltration of marrow and spleen by abnormal cells. No human immunoglobulin was detected in the sera from these 7 mice.

Five mice were inoculated with $3 \cdot 7 \times 10^6$ myeloma marrow cells from donor 3M which showed the characteristics of an IgA myeloma. All 5 mice were killed when sick or moribund 13 days after inoculation. No cells resembling plasma or myeloma cells were seen in the blood of any of these animals; infiltration by abnormal cells was found in the marrow in 4 and in the spleen in all 5. It was interesting to note that some of the plasma cells possessed " flaming " cytoplasm, an appearance said to be characteristic of human IgA myeloma. No human immunoglobulin was detected in the sera from these 5 mice.

Of 7 mice inoculated with $2 \cdot 2 \times 10^6$ myeloma marrow cells (donor 4M), 5 died unexpectedly 13 days after inoculation and 2 were killed when moribund on the 14th day. Peripheral blood smears from both these mice were normal but in each the marrow and spleen was infiltrated by abnormal cells. Human immunoglobulin (IgA, < 5 mg/100 ml) was detected in the serum from each of these mice.

Of 8 mice inoculated with $3{\cdot}1 \times 10^6$ myeloma marrow cells (donor 5M), 1 died and 6 were killed when sick or moribund 11 days after inoculation. Abnormal cells were found in the blood and marrow of 4 mice and in the spleens of all 6 (Fig. 2). In the serum from 5 of these mice human IgG (16–60 mg/100 ml), and in 1 human IgA (27 mg/100 ml), were detected.

The 11 mice receiving $2{\cdot}9 \times 10^6$ and $3{\cdot}4 \times 10^6$ myeloma marrow cells from donors 6M and 8M respectively showed no sickness and were killed 13 days after inoculation. Abnormal cells were present in the blood of 3 and in the marrow and the spleens of all 11 mice. The serum of 7 of these 11 mice showed the presence of human IgG (19, 19 and 21 mg/100 ml and in 4, < 5 mg/100 ml).

*First passage of abnormal mouse marrow and spleen cells originating from donors 2MA, 3M, 4M, 5M and 6M*

The 5 mice receiving $6{\cdot}6 \times 10^6$ intact abnormal mouse marrow cells originating from donor 2M (2MA) were killed when sick or moribund 12 days after inoculation. Peripheral blood smears were not examined but in all 5 mice the marrow and spleens were infiltrated by abnormal cells. Human immunoglobulin was not detected in the sera from these 5 mice.

Four mice received $1{\cdot}2 \times 10^6$ intact pooled abnormal mouse marrow and spleen cells (3M). One mouse was killed when sick 9 days later. The peripheral blood, marrow and spleen were heavily

FIG. 2.—Marrow film to show several plasma cells (arrowed P) and primitive cells (arrowed M) from mouse injected with myeloma marrow cells from donor 5M. May–Grünwald–Giemsa × 915.

infiltrated by abnormal cells but human immunoglobulin was not detected in the serum.

Six mice received $2 \cdot 5 \times 10^6$ intact pooled abnormal mouse marrow and spleen cells (4M). Although not apparently sick, these 6 mice were killed 21 days after inoculation. In 2 the spleens and blood smears, and in 1 the marrow, showed abnormal cells. No human immunoglobulin was detected in the serum from these 6 mice.

Of 5 mice receiving $2 \cdot 7 \times 10^6$ intact pooled abnormal mouse marrow and spleen cells (5M) 1 died and 4 became sick and were killed 10 days after inoculation. Peripheral blood smears from each of these 4 mice showed the presence of abnormal cells; such cells were found in the marrow and spleens of 3. Human immunoglobulins were not detected in the sera from these 4 mice. The 5 receiving $6 \cdot 1 \times 10^6$ intact pooled abnormal mouse marrow and spleen cells (6M) did not appear sick, but were killed 16 days after inoculation. In 3 the marrow, and in 1 the spleen and peripheral blood smear, showed the presence of abnormal cells. In the serum from 1 of these mice, human IgG (42 mg/100 ml) and human IgA ($< 5$ mg/100 ml) were detected.

*Second and third passage of abnormal intact mouse marrow and spleen cells originating from donor 5M*

The 5 mice receiving a second passage of pooled abnormal mouse marrow and spleen cells ($9 \cdot 4 \times 10^6$) became sick and were killed 11 days after inoculation. In 4, peripheral blood smears and marrow, and in all 5 the spleen, contained abnormal cells. No human immunoglobulin was detected in the sera from these 4 mice.

The 5 mice receiving a third passage of pooled intact mouse marrow and spleen cells ($3 \cdot 3 \times 10^6$) became sick and were killed 12 days after inoculation. In 4, peripheral blood smears showed the presence of abnormal cells, and the marrow and spleens were heavily infiltrated by abnormal cells in all 5. In the serum from 1 of these 5 mice, human immunoglobulin (IgG 23 mg/100 ml) was detected.

*Findings in surviving mice killed after a prolonged interval following inoculation*

The results following inoculation of intact human myeloma marrow cells in mice which had been prepared by adolescent thymectomy and receiving 900 rad followed by syngeneic mouse marrow cell replacement (T/900 rad), with or without the addition of ALS, and in surviving mice prepared by adolescent thymectomy and receiving 600 rad without syngeneic mouse bone marrow cell replacement (T/600 rad) are given in Table II. Of 4 mice (T/900 rad and BM) killed 12 months after receiving myeloma marrow cells from donor 1M, none showed abnormal cells in peripheral blood smears. In 2, abnormal cells were present in the marrow and spleen. No human immunoglobulin was detected in the serum of these 4 mice. Neither of the 2 mice (T/900 rad and BM and ALS) killed 11 months after receiving myeloma marrow cells from donor 1M showed abnormal cells in peripheral blood smears but in 1 mouse abnormal cells were present in marrow and spleen. Human immunoglobulin was not detected in the serum from either mouse. Of 4 mice (T/900 rad and BM) killed 7 months after receiving intact human myeloma marrow cells from donor 2M, 1 showed abnormal cells in peripheral blood smears and in 1 the marrow and spleen were infiltrated by abnormal cells. No human immunoglobulin was detected in the sera from these 4 mice. Of 3 mice (T/900 rad and BM and ALS) killed 7 months after receiving intact myeloma marrow cells from donor 2M, 1 had abnormal cells in the spleen; this mouse also showed lymphoid infiltration of the portal tracts of the liver. No human immunoglobulin was detected in the sera from these 3 mice.

Of 3 mice killed 5 months after surviving a first passage of pooled intact abnormal mouse marrow and spleen cells

FIG. 3.—Smear of spleen to show " flaming " cytoplasm in a plasma cell from an apparently healthy surviving mouse killed 5 months after intravenous inoculation with a first passage of pooled mouse marrow and spleen cells originating from donor 3M (IgA myeloma). May–Grünwald–Giemsa × 1350.

originating from donor 3M, no abnormal cells were found in peripheral blood smears or bone marrow, but in 1 the spleen showed an excess of plasma cells and there was lymphoid infiltration of the portal tracts of the liver. Some of the plasma cells again exhibited " flaming " cytoplasm (Fig. 3). Human immunoglobulin (IgG 120 mg/100 ml; IgA 31 mg/100 ml) was detected in the serum from this mouse.

Finally, 3 mice were killed 15 months after receiving intact myeloma marrow cells from donor 7M. None showed abnormal cells in peripheral blood smears, but in 2 the bone marrow and spleens showed the presence of abnormal cells. No human immunoglobulin was detected in the sera from these 3 mice.

### DISCUSSION

These experiments show that mice prepared by adolescent thymectomy followed by total body irradiation (600 rad) can be used to provide suitable conditions for the study of human myelomatosis and other haematological malignancies (Mitchell, Rees and Salsbury, 1972). Thus, within 10 to 30 days following the inoculation of intact human myeloma marrow cells a considerable proportion of mice so prepared yielded cytological and serological changes closely similar to those found in the human donor. These findings presumably result from the initial acceptance of a graft of human malignant cells and their subsequent replication within the immunologically deficient mouse model. Our findings in mice killed after a short interval following inoculation are closely similar to those of Phillips and Gazet (1970), who found that progressive growth of transplantable human carcinoma cell lines would grow for at least one month in ALS treated mice which had previously been thymectomized. Similarly, Berenbaum (1971) transplanted human tumours into mice which had been thymectomized, irradiated and treated with antilymphocyte serum; about one third of the tumours grew for several weeks, with retention of the histological appearances seen in man.

The possibility that the histological changes found in mouse bone marrow or spleen might result from adolescent thymectomy or total body irradiation irrespective of the inoculation of human myeloma marrow cells, has been critically examined. Thus, among identically prepared but uninoculated mice from the same stock which served as collateral controls to mice receiving human myeloma marrow cells, the marrows were hypoplastic but showed no infiltration by abnormal cells when examined after similar intervals. The spleens of these uninoculated mice showed active erythropoiesis, but in none was there evidence of infiltration by abnormal cells; plasma cells were occasionally seen but when present were few in number and widely scattered. Similarly, the marrows and spleens of all mice identically prepared but receiving normal human bone marrow cells or a first passage of pooled mouse bone marrow and spleen cells originating from these mice, showed evidence of active erythropoiesis and the splenic appearances were closely similar to those described by Till and McCulloch (1961).

The presence of human immunoglobulin (IgG and IgA), as detected by immunodiffusion of the undiluted sera of mice 11 to 17 days following the inoculation of intact human myeloma marrow cells, might be attributable to the transfer of human serological components in the inoculum. This explanation could account entirely for the serological findings in these mice; it is relevant that traces of human IgG and IgA were also found in the sera of mice showing no infiltration by abnormal cells and which were bled after similar intervals following the inoculation of intact and normal human marrow cells.

Cytological changes largely indistinguishable from those present in the human donor were found in the marrow, spleen and peripheral blood of a substantial proportion of mice receiving a first, second or a third passage of abnormal mouse marrow and spleen cells originated from no less than 5 separate human donors with myelomatosis. Moreover, human immunoglobulin (IgG 42 mg/100 ml; IgA, $<$ 5 mg/100 ml) was detected in the serum of one mouse receiving a first passage of abnormal mouse marrow and likewise in the serum of one mouse receiving a third passage of abnormal mouse marrow and spleen cells originating from a different human myeloma donor. These mouse sera have been examined against a number of class specific antisera and the presence of human immunoglobulin has been confirmed. Similarly, it is of interest that a proportion of apparently healthy surviving mice receiving intact human myeloma marrow cells from each of 3 separate donors, again showed the presence of changes in marrow, spleen or peripheral blood closely similar to those seen in the human donor when killed 7 to 15 months after inoculation. The findings in one other of these mice remaining apparently healthy after receiving a first passage of abnormal mouse marrow and spleen cells originating from donor 3M (IgA myeloma), are of especial interest. When killed 5 months after inoculation this mouse showed abnormal cells in the spleen; some of the plasma cells seen exhibited flaming cytoplasm (Fig. 3). Again, human immunoglobulin (IgG 120 mg/100 ml; IgA 31 mg/100 ml) was detected in the serum from this mouse.

These findings certainly provide evidence of the ability of mice so prepared to support a graft of human myeloma marrow cells. Although various lines of human tumour cells have been established which are indefinitely transplantable in immunosuppressed animals, the transplantation of tumours freshly obtained from patients is much less successful. For example, Phillips and Gazet (1970) found that only 12 of 66 human tumours which they attempted to transplant to thymectomized ALS treated mice, appeared viable 25 days after transplantation. It would therefore seem unlikely that our findings can be accounted for entirely by the persistence and multiplication of the injected human cells, although we have been unable to find any previous account of the inoculation of human myeloma marrow cells in the experimental animal.

An alternative explanation might be the possibility that cell hybridization may occur between human myeloma marrow and normal mouse cells, thus giving rise to other cells secreting myeloma protein. Such *in vivo* hybridization would be similar to that described by Wiener, Klein and Harris (1973). In this context, the findings of Schwaber and Cohen (1973), who showed by cell culture that a hybrid clone resulting from the fusion of a myeloma mouse cell with a human lymphocyte (initially shown to produce no immunoglobulin) subsequently secretes both human and mouse immunoglobulins, are highly relevant. Finally, there remains the possibility that in a proportion of the immunologically deficient mice in our experiments the mouse marrow cells became infected by an agent from the human myeloma marrow cells following their initial acceptance as a graft (Mitchell, Rees and Salsbury 1971, 1972).

Further detailed studies designed to establish more precisely the most likely explanation for our present findings are currently in progress.

### REFERENCES

Berenbaum, M. C. (1971) *Growth of Human Neoplasms in Immunosuppressed Mice.* Research Unit's Reports p[1], Leukaemia Research Fund Annual Report.

Handler, A. H., Davis, S. & Sommers, S. C. (1956) Heterotransplantation Experiments with Human Cancers. *Cancer Res.*, **16**, 32.

Kaufman, J. J. & Lichtenauer, P. (1967) Experimental Studies of Human Bladder Cancer: Heterotransplantation to the Hamster Cheek Pouch. *Br. J. Urol.*, **39**, 490.

Lance, E. M. & Medawar, P. B. (1968) Survival of Skin Heterografts under Treatment with Antilymphocytic Serum. *Lancet*, i, 1174.

Mitchell, D. N., Rees, R. J. W. & Salsbury, A. J. (1971) Possible Transmissibility of Human Myelomatosis in Immunologically Deficient Mice. *Lancet*, ii, 1009.

Mitchell, D. N., Rees, R. J. W. & Salsbury, A. J. (1972) Leukaemic Transformation of Grafted Marrow. *Lancet*, ii, 179.

Patterson, W. B., Patterson, H. R. & Chute, R. N. (1957) Transplantable Human Tumors. *Cancer, N.Y.*, **10**, 1281.

Phillips, B. & Gazet, J. C. (1967) Growth of Two Human Tumour Cell Lines in Mice Treated with Antilymphocyte Serum. *Nature, Lond.*, **215**, 548.

Phillips, B. & Gazet, J. C. (1968) Effect of Antilymphocyte Serum on the Growth of Hep 2 and HeLa Cells in Mice. *Nature, Lond.*, **220**, 1140.

Phillips, B. & Gazet, J. C. (1970) Transplantation of Primary Explants of Human Tumour to Mice Treated with Antilymphocyte Serum. *Br. J. Cancer*, **24**, 92.

Schwaber, J. & Cohen, E. P. (1973) Human X Mouse Somatic Cell Hybrid Clone Secreting Immunoglobulins of Both Parental Types. *Nature, Lond.*, **244**, 444.

Sheard, C. E. Double, J. A. & Berenbaum, M. C. (1971). The Sensitivity to Chemotherapeutic Agents of a Rat Tumour Grown in Immunosuppressed Mice. *Br. J. Cancer*, **25**, 838.

Till, J. E. & McCulloch, E. A. (1961) A Direct Measurement of the Radiation Sensitivity of Normal Mouse Bone Marrow Cells. *Radiat. Res.*, **14**, 213.

Toolan, H. W. (1953) Growth of Human Tumours in Cortisone-treated Laboratory Animals: the Possibility of Permanently Transplantable Human Tumors. *Cancer Res.*, **13**, 389.

Toolan, H. W. (1958) VI. The Human Tumor in Heterologous Hosts. The Transplantable Human Tumor. *Ann. N.Y. Acad. Sci.*, **76**, 733.

Wiener, F., Klein, G. & Harris, H. (1973) The Analysis of Malignancy by Cell Fusion. IV. Hybrids between Tumour Cells and a Malignant L Cell Derivative. *J. cell Sci.*, **12**, 253.